# 5-Azacytidine Suppresses the Expression of Tissue-Specific Oct-1 Isoform in Namalwa Burkitt’s Lymphoma Cell Culture

**DOI:** 10.1134/S1607672922020089

**Published:** 2022-05-10

**Authors:** A. P. Kotnova, A. G. Stepchenko, Yu. V. Ilyin, S. G. Georgieva, E. V. Pankratova

**Affiliations:** Engelhardt Institute of Molecular Biology of Russian Academy of Sciences, Moscow, Russia

**Keywords:** transcription factor POU2F1 (Oct-1), alternative promoters, 5-azacytidine, Burkitt’s lymphoma

## Abstract

Overexpression of the transcription factor POU2F1 (Oct-1) increases the malignant potential of the tumor and determines the unfavorable prognosis for both solid and hematological cases of the disease in human carcinogenesis. The Oct-1 level determines the rate of development of the disease in acute myelodysplastic leukemia (AML), and a decrease in its expression significantly delays the development of leukemia in mice; however, a complete knockout of Oct-1 leads to the death of the animals. POU2F1 (Oct-1) is expressed as several isoforms transcribed from alternative promoters. They include both ubiquitous and tissue-specific isoforms. It was shown that in Burkitt’s lymphoma Namalwa cells 5-azacytidine specifically suppresses the expression of the tissue-specific isoform Oct-1L mRNA (level of Oct-1L is abnormally increased in these cells), while not causing changes in the amount of the ubiquitous isoform Oct-1A mRNA. These results show that it is possible to selectively reduce the transcription level of the Oct-1L isoform aberrantly expressed in human tumor cells.

Malignant tumors of hematopoietic and lymphoid tissue account for approximately 8% of all malignant diseases. They are divided into two large groups—lymphomas and leukemias; many of them are characterized by poor prognosis and low survival rate. The search for markers and therapeutic targets for personalized treatment of oncological diseases showed that the product of the *POU2F1* (*Oct-1*) gene is an extremely significant factor in the development and progression of many malignant tumors of both epithelial origin and tumors of the hematopoietic and lymphoid tissues [1].

The transcription factor Oct-1 belongs to the family of POU transcription factors with a highly conserved DNA-binding domain and controls the differentiation, survival, and proliferation of immune system cells and hematopoietic cells [2, 3]. Oct-1 is expressed in all cells of the body, regulates the differentiation of B and T cells and hematopoietic stem cells [2, 3], is involved in protecting cells from various types of stresses (genotoxic, oxidative, hypoxic, and endoplasmic reticulum stress), and modulates the response of the cell for chemotherapy drugs [4, 5].

An increase in the level of Oct-1 expression in tumor cells significantly contributes to an unfavorable prognosis for the development of oncological diseases. For example, determining the expression level of POU2F1 (Oct-1) in gastric cancer is of an even higher prognostic value than determining the stage (I–IV) of the disease according to AJCC [6]. For tumors of the hematopoietic system, the pro-oncogenic functions of Oct-1 have been described for Hodgkin’s lymphoma, thymus lymphoma, diffuse large B-lymphoma, and acute myeloid leukemia [7–9]. Overexpression of Oct-1 is often observed in diffuse large B-cell lymphoma and is an independent predictor of poor outcome [8]. Oct-1 is an important regulator of leukemogenicity and hematopoietic stress. A high expression level of the transcription factor Oct-1 protects hematopoietic cells from stress but promotes the development of thymic lymphoma [1] and acute myeloid leukemia [9] in mice. In contrast, downregulation of Oct-1 protects mice from leukemia induced by the MLL–AF9 fusion oncoprotein. The combination of this AML model system with Oct-1 knockout showed that the loss of one Oct-1 allele significantly delayed the development of leukemia. Deletion of both Oct-1 alleles completely protects mice from leukemia but leads to bone marrow failure and death of animals [9]. Analysis of these data indicates that Oct-1 is a potent factor that determines the malignant potential of a tumor and its response to chemotherapy drugs.

The polyfunctionality of Oct-1 is largely determined by the fact that it exists in the cell as a number of different isoforms that are formed due to alternative splicing and/or alternative transcription initiation [10]. There are alternative promoters in the *POU2F1* gene [10–12]. As can be seen from Fig. 1, the transcripts read from them have different first exons and encode isoforms that differ in their N-terminal sequences [11]: the Oct-1A ubiquitous isoform is read from the U promoter, whereas the tissue-specific Oct-1L and Oct-1R isoforms are read from the L promoter.

**Fig. 1.**
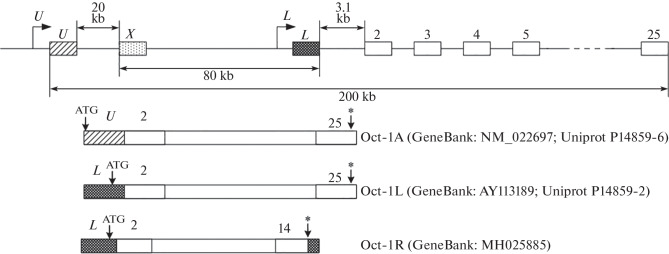
Exon–intron organization of the *POU2F1* (*Oct-1*) gene. The structure of isoforms transcribed from the ubiquitous U-promoter and the tissue-specific L-promoter is shown. Exons are indicated by rectangles. Alternative 5'-end exons are indicated by shading. The start of transcription is shown with turning arrows. The translation starts and the stop codons are indicated by arrows and asterisks, respectively.

The expression of Oct-1 isoforms changes during hematopoietic cell differentiation: in pluripotent CD34+ hematopoietic cells (PHC), Oct-1L is expressed at a high level; its expression level drastically decreases during the differentiation of T cells (CD3+) and monocytic cells (CD14+) but almost does not change during the differentiation of B cells (CD19+) [13, 14]. The human Oct-1R isoform is expressed only in B cells and was not found in PHCs [13]. Characteristically, the level of the ubiquitous Oct-1A isoform does not undergo significant changes during the differentiation of hematopoietic cells.

In all normal hematopoietic cells, the activity of the tissue-specific L promoter and, therefore, the concentration of the tissue-specific isoforms is lower than the activity of the ubiquitous U promoter and the concentration of the ubiquitous Oct-1A isoform. In Burkitt’s B-cell lymphomas Namalwa and Raji, this ratio is disturbed, and the concentration of the L isoform significantly exceeds the content of the A isoform in cells. The concentration of the tissue-specific Oct-1L isoform in Namalwa B cells is several times higher than in the normal B cells (CD19+) [13]; its expression level is also increased in the Jurkat T cell line as compared to the normal T cells (CD3+) [13]. Notably, all these cell lines were originally derived from poorly differentiated lymphoblasts.

Previously, it was shown that overexpression of Oct-1R and Oct-1L in Namalwa cells leads to repression of many genes involved in the differentiation of B and T cells, as well as monocytic cells (CD14+) [13, 14]. The high level of the Oct-1L isoform observed in lymphoblastic tumor cell lines indicates that an excess of Oct-1L, apparently, significantly reduces their ability to differentiate. The existence of alternative promoters in the *POU2F1*(*Oct*-*1*) gene makes it possible to influence not only the expression of total Oct-1, which has a detrimental effect on the body, but also the expression of its individual isoforms, the level of which is increased in tumor cells.

In this study, we investigated the effect of 5-azacytidine on the transcription of the *POU2F1* gene and showed that it selectively suppresses the transcription from the tissue-specific L promoter of the *POU2F1* gene and reduces the concentration of the Oct-L isoform in tumor cells of Namalwa Burkitt’s lymphoma.

The DNA methylation inhibitor 5-azacytidine is used in clinical practice to treat myelodysplastic syndrome (MDS) and acute myeloid leukemia (AML). To study its effect on the level of transcription of alternative Ost-1 protein isoforms, Namalwa cells were seeded into 6-well plates (2 × 10^6^ per well) in DMEM medium   supplemented   with   10%   fetal   serum. 5-Azacytidine (Sigma-Aldrich) dissolved in 8 µL of DMSO was added to the cells at concentrations 10, 5, 2.5, and 1.25 µM; 8 μL of DMSO was added to the control wells. To assess the effect of 5-azacytidine on the level of transcription of different Oct-1 isoforms, RNA was isolated from the cell culture by the trizol method. Then, reverse transcription was performed using the Maxima First Strand cDNA Synthesis Kit for RT-qPCR (Thermo Scientific), and real-time PCR was performed using primers specific to A and L isoforms (Oct-1A-sense: 5'-tattcaaaatggcggacgga-3'; Oct-1L-sense: 5'-ccaccccaaactgctacctgt-3'; Oct-1-antisense, common for both isoforms: 5 '-ctgacggattgttcattcttgagt-3'). Normalization was performed with respect to the *GUS* gene (GUS sense 5'-cgtggttggagagctcatttgga-3' and GUS antisense 5'-attccccagcactctcgtcggt-3').

As can be seen from Fig. 2, when Namalwa cells were cultured for 24 h in a medium containing 5-azacytidine, the amount of the Oct-1L isoform transcribed from the alternative tissue-specific L promoter significantly decreased, whereas the amount of mRNA of the ubiquitous Oct-1A isoform remained virtually unchanged. This effect was dose-dependent and manifested itself at the level of concentrations used  in  clinical  practice.  In  the  presence  of  10 µM 5-azacytidine in the culture medium, the amount of Oct-1L mRNA in Namalwa cells decreased 3 times compared with the control, and in the presence of 5 µM 5-azacytidine it decreased twice. A further decrease in the concentration of 5-azacytidine did not cause significant changes in the Oct-1L expression.

**Fig. 2.**
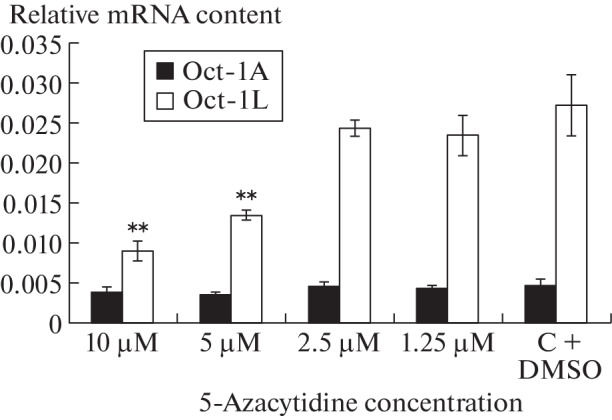
Effect of 5-azacytidine on the transcription level from the alternative U and L promoters of the *POU2F1* (*Oct-1*) gene. Quantitative PCR results. The chart represents the mean value ±SEM for three independent experiments; *t* tests were performed to determine whether the difference between the mean values for the control and treated cells was significant (** *p* < 0.01).

The presented results indicate that 5-azacytidine inhibits the transcription from the tissue-specific L promoter, reducing the concentration of the tissue-specific isoforms in tumor cells, but has almost no effect on the transcription from the ubiquitous U promoter and on the concentration of mRNA of the ubiquitous isoform A.

At low concentrations, which are currently used in oncohematological practice, 5-azacytidine serves as a hypomethylating agent that inhibits DNA methyltransferase by incorporating azacitidine triphosphate into DNA. This leads to loss of DNA methylation and reactivation of repressed genes. It is believed that pathological DNA methylation patterns determine the development of high-risk myelodysplastic syndromes and acute myelocytic leukemia, and hypomethylation can restore the normal function of genes that control differentiation and proliferation [15–17]. After 16 years of clinical use, 5-azacytidine remains the key drug used for treatment for MDS and AML. However, the exact mechanism of its action is still not fully understood [17]. In our study, we have shown that 5-azacytidine suppresses the transcription from the tissue-specific L promoter of the *POU2F1* gene and selectively reduces the expression of the Oct-1L isoform 2–3 times at concentrations used in clinical practice.

We analyzed the region of the alternative L promoter of the *POU2F1* gene and found no CpG islands there that could be methylated. Therefore, we believe that the discovered effect of 5-azacytidine is not associated with the demethylation of the L promoter. Possibly, more distant regions of the *POU2F1* gene regulating the transcription of the tissue-specific promoter undergo demethylation, or other properties of 5-azacytidine that are not associated with demethylation are manifested.

In the treatment of oncological diseases, attention should be paid to the following facts: (1) a high level of Oct-1 expression complicates the course of the disease in hematological tumors such as AML, diffuse large B-cell lymphoma, Hodgkin’s lymphoma, and thymus lymphoma; (2) artificially induced hypoexpression of Oct-1 in AML delays the development of leukemia; (3) B- and T-cell lymphomas (Namalwa, Raji, and Jurkat cell lines) are characterized by an abnormally high expression of the Oct-1L isoform, which is uncharacteristic of normal lymphoid cells; (4) in some tumor cell lines, the expression level of the Oct-1L isoform significantly exceeds that in the normal cells of the same origin [18]. Taken together, these data led us to conclude that the aberrant expression of the tissue-specific Oct-1L isoform is an unfavorable prognostic factor and can become a therapeutic target for tumors with a high level of Oct-1. In these cases, it is possible to use 5-azacytidine, which selectively suppresses the expression of the Oct-1L isoform, in combination therapy. This assumption requires further analysis of the expression of Oct-1 isoforms in primary tumors.

It is known from clinical practice that monotherapy with 5-azacytidine is effective in the treatment of MDS and AML. However, there is currently insufficient evidence to support the use of 5-azacytidine in the treatment of solid tumors or other hematological malignancies. The only attempt to use 5-azacytidine for the treatment of solid tumors was made in 1977. An antitumor effect was observed only in 17% of the examined patients with breast carcinoma and in 21% of patients with malignant lymphomas. As a result, it was concluded that the drug is ineffective for solid tumors [15]. However, recent studies have shown that tumors should be divided according to the level of Oct-1 expression. Overexpression of Oct-1 correlates with tumor aggressiveness in cancers of the breast, esophagus, stomach, prostate, lung, head and neck, cervix, colorectal cancer, diffuse large B-cell lymphoma, and other malignant tumors [1, 8]. Possibly, the positive antitumor effect of 5-azacytidine in the 1977 study was observed precisely in the patients with overexpression of Oct-1L in the tumor.

Our results support the importance of detailed gene expression analysis as a strategy for identifying new biomarkers and therapeutic targets in lymphomas and leukemias. The ability of 5-azacytidine to suppress the Oct-1L expression in tumor cells may be an important step for its use in the treatment of tumors with increased Oct-1L expression.
